# Obituary

**Published:** 1909-10-23

**Authors:** 


### Obituary.

We have to record the death of Dr. John Pringle, at his house at Hampstead. Dr. Pringle, who was 68, took his degree of M.D. at Edinburgh in 1863, becoming L.R.C.S. Edin. in the same year. Later he studied in Dublin, taking his L.M. at the Rotunda Hospital in 1870. Some years ago he was medical officer of health for Strathmiglo, Fife.

On October 13, at The Moats, Thame, Herbert Grove Lee, M.D., aged 69. Dr. Lee graduated at St. Andrews in 1861, and in the same year took his M.R.C.S. and L.S.A.

Dr. William Clibborn died suddenly at 12 Bolton Gardens, W., last week. He graduated at Trinity College, Dublin, in 1875, and was Medical Officer of Health for Bridgport, Medical Superintendent Bridgport Isolation Hospital, and Honorary Surgeon to the Birmingham Lying-in Charity.

The death is announced in Bath of Mrs. Harriet Newcomb, the elder daughter of the late Edward Taylor Meredith, M.R.C.S., L.S.A.

Dr. Robert Lindsay, M.A., M.B., F.R.C.S.E., late Army Medical Department, died on the 15th inst. at his residence, The Oaks, Betley, Hampshire.

Dr. James Irvine Menzies, æt. 75, at 47 Earl's Court Square. Dr. Menzies was an old student of St. Bartholomew's Hospital, and qualified in 1859.

The death of Dr. John Herbert Wells, at Ditchling, in Sussex, is announced at the age of thirty. He studied at St. Mary's Hospital and obtained his M.B. and B.S. degrees at the University of London with honours in medicine and pathology at the University. He was a member of the Royal College of Surgeons and a Licentiate of the Royal College of Physicians, a member of the Harveian and Pathological Societies, a Fellow of the Royal Society of Medicine, and formerly held the posts of house physician and demonstrator on histology, physiology, and pathology at St. Mary's Hospital. In the year 1901 he was selected as scientific assistant to the special chloroform committee of the British Medical Association.

